# Predicting loss of independence and mortality in frontotemporal lobar degeneration syndromes

**DOI:** 10.1136/jnnp-2020-324903

**Published:** 2021-02-09

**Authors:** Alexander G Murley, Matthew A Rouse, Ian T S Coyle-Gilchrist, P Simon Jones, Win Li, Julie Wiggins, Claire Lansdall, Patricia Vázquez Rodríguez, Alicia Wilcox, Karalyn Patterson, James B Rowe

**Affiliations:** 1 Clinical Neurosciences, University of Cambridge, Cambridge, UK; 2 Neurology, Cambridge University Hospitals NHS Foundation Trust, Cambridge, UK; 3 Neurology, Norfolk and Norwich University Hospitals NHS Foundation Trust, Norwich, UK; 4 MRC Cognition and Brain Sciences Unit, University of Cambridge, Cambridge, UK

## Abstract

**Objective:**

To test the hypothesis that in syndromes associated with frontotemporal lobar degeneration, behavioural impairment predicts loss of functional independence and motor clinical features predict mortality, irrespective of diagnostic group.

**Methods:**

We used a transdiagnostic approach to survival in an epidemiological cohort in the UK, testing the association between clinical features, independence and survival in patients with clinical diagnoses of behavioural variant frontotemporal dementia (bvFTD n=64), non-fluent variant primary progressive aphasia (nfvPPA n=36), semantic variant primary progressive aphasia (svPPA n=25), progressive supranuclear palsy (PSP n=101) and corticobasal syndrome (CBS n=68). A principal components analysis identified six dimensions of clinical features. Using Cox proportional hazards and logistic regression, we identified the association between each of these dimensions and both functionally independent survival (time from clinical assessment to care home admission) and absolute survival (time to death). Analyses adjusted for the covariates of age, gender and diagnostic group. Secondary analysis excluded specific diagnostic groups.

**Results:**

Behavioural disturbance, including impulsivity and apathy, was associated with reduced functionally independent survival (OR 2.46, p<0.001), even if patients with bvFTD were removed from the analysis. Motor impairments were associated with reduced absolute survival, even if patients with PSP and CBS were removed from the analysis.

**Conclusion:**

Our results can assist individualised prognostication and planning of disease-modifying trials, and they support a transdiagnostic approach to symptomatic treatment trials in patients with clinical syndromes associated with frontotemporal lobar degeneration.

## Introduction

Prognosis in syndromes associated with frontotemporal lobar degeneration (FTLD) is highly variable and difficult to predict. Disease duration is not fully explained by the standard diagnostic categorisation into behavioural variant frontotemporal dementia (bvFTD), non-fluent (nfvPPA) or semantic (svPPA) variants of primary progressive aphasia, progressive supranuclear palsy (PSP) or corticobasal syndrome (CBS).^1–4^ Better prognostic models would aid both trial design and clinical management.

The syndromes caused by FTLD have highly heterogeneous and overlapping clinical features.^5–7^ Our hypothesis was that a subset of clinical features, represented across the spectrum of disorders, explains variation in functional independence and survival. We therefore adopted a transdiagnostic approach, increasingly used in psychiatric and neurological diseases, to identify prognostic clinical features across the FTLD syndrome spectrum.^8 9^ Previous work has identified that features of motor neuron disease reduce the prognosis in bvFTD,^3 10^ while dysphagia and cognitive impairment worsen prognosis in PSP-Richardson’s syndrome.^11^ Here, we focus on cognitive, behavioural and motor features of disease. Mortality is a definite endpoint in FTD, PSP and CBS. However, these disorders also engender dependency and caregiver burden.^4 12^ Community-based studies suggest that increased dependency, from cognitive or physical impairment, predicts care home admission.^13 14^ We used care home admission as a disease endpoint that indirectly represents loss of functional independence.^4 15^ Care home admission is not a direct measure of independence, as residents may remain independent with activities of daily living, whereas a patient at home might be very dependent. However, at the group level, care home admission can provide insights into the impact of disease on independence, and it is a definite endpoint of interest to patients and their families. Behavioural impairment is a risk factor for care home admission in patients with dementia,^16^ while patients with PSP and CBS, on average, have a worse prognosis than those with bvFTD or PPA.^3^ This distinction led to the hypothesis that behavioural impairments and motor impairments contribute to the risk of care home admission and mortality, respectively.

Given the heterogeneity *within* each of the syndromes associated with FTLD,^6 17^ and phenotypic overlap *between* syndromes,^5 7^ we proposed that the presence of clinical features would predict prognosis over and above the diagnostic group. Combinations of clinical features were identified by principal components analysis, rather than the diagnostic labels, overcoming some of the limitations of categorical clinical diagnostic criteria.

## Methods

### Participant recruitment and clinical review

**Figure 1:**
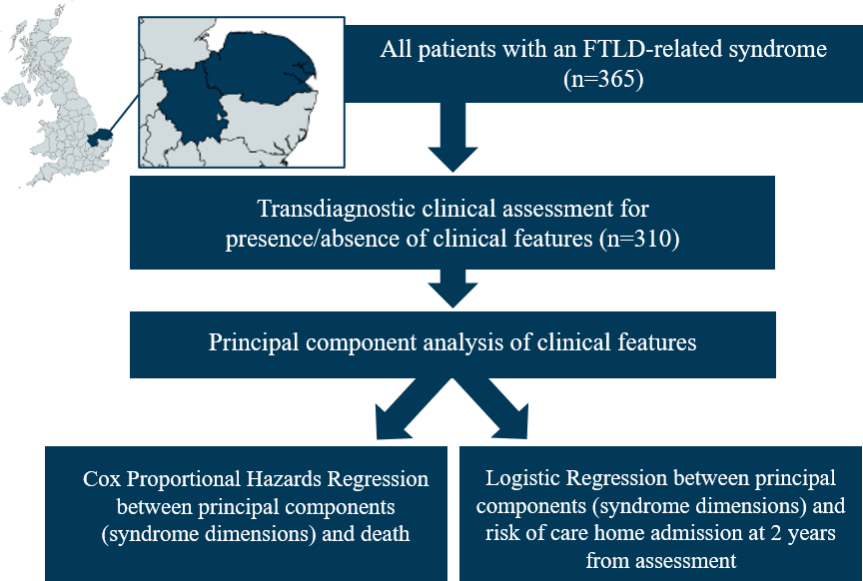
Diagram of study methods. Three hundred ten out of 365 patients in the study catchment area had a clinical assessment. A principal component analysis of the clinical features across all frontotemporal lobar degeneration (FTLD) syndromes yielded six components. We tested the association between these components and mortality (using Cox proportional hazards regression) and risk of care home admission (using logistic regression).

Survival data were collected for all participants in the PIPPIN study (Pick’s disease and Progressive Supranuclear Palsy Prevalence and Incidence), a cross-sectional epidemiological study, details of which have been previously reported.^2 7^ This study enrolled, via multisource referral, all patients with a designated syndrome associated with FTLD living in the UK counties of Cambridgeshire and Norfolk over two 24-month periods (2013–2014, 2017–2018) (figure 1).

We use current consensus terminology: frontotemporal lobar degeneration (FTLD) refers to neuropathological classification. Such FTLD is associated with a range of clinical syndromes that include behavioural variant frontotemporal dementia (bvFTD),^18^ non-fluent (nfvPPA) and semantic (svPPA) variants of primary progressive aphasia,^19^ progressive supranuclear palsy (PSP)^6^ and corticobasal syndrome (CBS).^17^ Patients with coexistent motor neuron disease (eg, ‘FTD-MND’) were included but not patients with motor neuron disease in isolation. We group all PSP subtypes into the PSP group.^6^ We also grouped patients with logopenic variant primary progressive aphasia and primary progressive aphasia who did not meet the criteria for one of the three PPA subtypes, noting that these latter groups commonly have underlying Alzheimer’s disease. Five patients had the combination of behavioural impairment meeting the diagnostic criteria for bvFTD, prosopagnosia and predominant right temporal lobe atrophy on neuroimaging.^20^ Rather than a separate ‘right semantic dementia’ or ‘right temporal lobe variant FTD’ group, we include these five cases in the bvFTD group. If a participant met the diagnostic criteria for more than one syndrome (eg, PSP and CBS), the clinical diagnosis label was based on judgement of the dominant clinical phenotype by the multidisciplinary team at the Cambridge University Centre for FTD or Cambridge University Centre for Parkinson plus. We assessed in person 310 of the 365 patients identified as alive and living in area in the ascertainment windows. A clinical, cognitive and language assessment recorded the presence or absence of clinical symptoms and signs included in the current diagnostic criteria for FTLD syndromes,^6 17–19^ plus cognitive assessment using the Addenbrookes Cognitive Examination—Revised (ACER) and carer interview using the Cambridge Behavioural Inventory—Revised (CBIR). The cross-sectional design of the study means that the clinical assessment occurred at diverse stages in patients’ disease course.

We recorded dates of care home admission and death from each participant’s NHS Summary Care Record. This database includes information on the address and date of death of every UK resident, minimising loss to follow-up. We defined a care home as an institution registered with the UK government to provide residential and/or nursing care. All participants provided written informed consent or, if they lacked capacity to consent, then their next of kin was consulted using the ‘personal consultee’ process according to the UK law.

### Statistical analysis

We employed a transdiagnostic, data-driven approach using principal component analysis to identify syndrome dimensions of covarying clinical features. Forty-five clinical features were combined into 24 groups of related features by summing the number of present features in each group.^7^ The clinical feature group scores, ACER and CBIR results were standardised into z scores and then entered into a principal component analysis. We identified six components using Cattell’s criterion and then performed varimax rotation.

We used a Cox proportional hazards regression analysis to test the association between these six clinical syndrome components and the time from clinical assessment to death (covariates of age, gender and disease group). This allows all participants to be included in the survival analysis, censoring participants who did not reach the endpoint (death). The predictor variables (subject weightings on each syndrome dimension) were z scored to aid interpretation. If a syndrome dimension closely resembled typical features of a specific diagnostic group, we repeated the Cox proportional hazards regression analysis without that group.

Next, we tested the association between the syndrome dimensions and time to care home admission using logistic regression, with the binary outcome of care home admission by 2 years from study assessment. Patients in a care home at study assessment or those with incomplete follow-up were excluded from this analysis. We used logistic rather than Cox proportional hazards regression because it could be argued that the risk of care admission does not remain constant over time (an assumption of Cox hazards regression). See figure 1 for a summary of the study methods.

All patients had a clinical assessment but the ACER and CBI-R were missing in a minority of participants (6.32% of the total dataset), which were imputed using trimmed scored regression^21^ using the partial dataset of that participant as predictors. All analyses were performed in Matlab V.2018b (Mathworks, USA). Kaplan-Meier curves were plotted using the *MatSurv* function (https://github.com/aebergl/MatSurv).

### Data availability

Anonymised derived data are available on reasonable request for academic purposes, subject to the protection of personally identifiable data.

## Results

Three hundred sixty-five patients with a designated diagnosis were identified as alive in region within the time windows, of whom 310 were assessed in person by the study team (bvFTD n=64, nfvPPA n=36, svPPA n=25, other PPA n=16, PSP n=101, CBS n=68). The epidemiological, phenotypic, neuropsychological and imaging characteristics of this cohort at baseline have been published previously.^2 7 22^ Summary demographic and survival results are shown in table 1. At the censor date (1 August 2019), 169 patients with FTLD (54.5%) had been admitted to a care home and 200 patients (64.5%) had died. Most patients were admitted to a care home before they died (131/200, 65.5%).

**Table 1:** Demographics of the study cohort

	All FTLD	bvFTD	nfvPPA	svPPA	PPA (lv/mixed)	PSP	CBS
Total in catchment area (n)	365	81	40	28	16	123	77
Clinical phenotyping (n)	310	64†	36‡	25	16	101	68
Age (mean years) (SD)	70.26 (8.57)	64.59 (9.56)	72.09 (8.81)	67.55 (6.43)	70.80 (7.05)	72.56 (7.14)	72.08 (7.69)
Gender (male/female)	152/158	33/31	15/21	14/11	7/9	56/45	27/41
Symptom onset to study assessment (years, mean and SD)	4.75 (3.18)	5.70 (4.45)	2.83 (1.93)	4.96 (2.69)	2.76 (1.97)	4.50 (2.94)	4.71 (2.77)
Diagnosis to study assessment (years, mean and SD)	1.44 (2.77)	1.88 (3.88)	1.09 (1.27)	1.65 (2.01)	1.58 (1.67)	1.02 (1.17)	1.73 (2.02)
Symptom onset to death (years, mean and SD)*	7.71 (4.37)	9.08 (7.00)	7.93 (3.47)	11.03 (3.39)	9.29 (3.14)	6.39 (3.67)	7.30 (3.12)
Diagnosis to care home (years, mean and SD)*	2.94 (2.43)	2.26 (2.90)	4.43 (1.75)	5.31 (1.86)	4.44 (2.48)	1.69 (1.20)	3.13 (2.28)
Diagnosis to death (years, mean and SD)*	4.40 (3.25)	5.49 (5.06)	5.50 (2.62)	7.95 (2.61)	5.74 (2.19)	2.78 (2.7)	4.12 (2.35)
Postmortem neuropathology	53	8	4	5	1	16	19
Pathology diagnoses		PiD=1 PSP=1 TDP=6	CBD=3 AD=1	PiD=1 TDP=4	AD=1	PSP=16	CBD=6 AD=8 Other=3

*Subgroup of cohort with complete follow-up. Six patients were living in a care home at diagnosis.

†Twelve patients with bvFTD had motor neuron disease.

‡One patient with nfvPPA had motor neuron disease.

AD, Alzheimer’s disease pathology; CBD, corticobasal degeneration; PiD, Pick’s disease pathology; PSP, progressive supranuclear palsy pathology; TDP, 43 kDa Tar DNA binding portein, TDP43 pathology.

**Figure 2:**
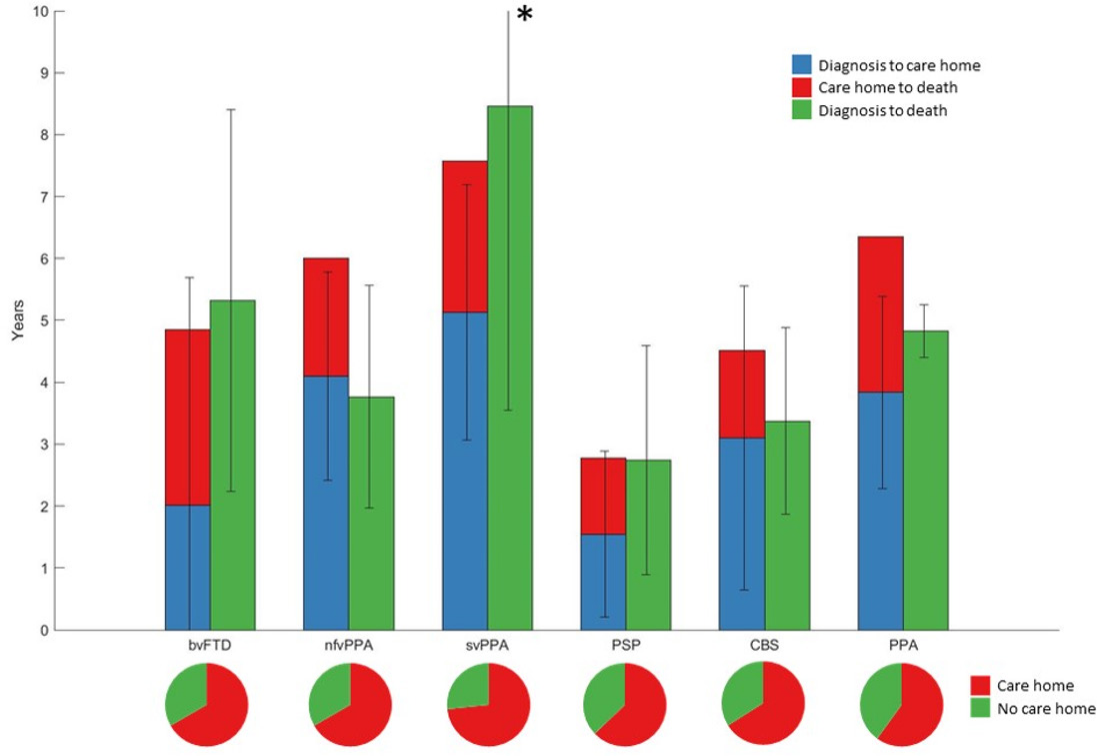
Survival in frontotemporal lobar degeneration syndromes. The bar plot shows disease duration in frontotemporal lobar degeneration (FTLD) syndromes in patients with complete follow-up from disease onset to death. *SD of svPPA diagnosis to death was 4.91 years. Survival in each FTLD subgroup is shown grouped by care home versus no care home admission. The pie charts show proportion of each FTLD subgroup admitted to a care home during the disease course. bvFTD, behavioural variant frontotemporal dementia; CBS, corticobasal syndrome; nfvPPA, non-fluent variant primary progressive aphasia; PPA, progressive aphasia; PSP, progressive supranuclear palsy; svPPA, semantic variant primary progressive aphasia.

There was high variability in the time from diagnosis to care home admission or death in all groups (figure 2). Life expectancy differed between groups (analysis of variance, F1,5=10.41, p<0.001). This was primarily due to longer life expectancy in svPPA compared with PSP (mean difference 5.24 years, p<0.001), CBS (3.83 years, p<0.001) and bvFTD (2.69 years, p=0.047). Patients with PSP also had a worse prognosis compared with bvFTD (mean difference 2.55 years, p<0.001) and nfvPPA (2.54 years, p<0.001). Thirteen patients with FTD-MND had a lower mean time between diagnosis and death than the whole bvFTD cohort (2.67 years vs 5.49 years). Post hoc tests were Bonferroni corrected.

Using principal component analysis, we identified six clinical symptom dimensions with Catell’s criteria (Kaiser-Meyer-Olkin=0.86) (table 2). An individual’s score on each dimension showed the extent to which they expressed that clinical phenotype. Note that the principal component analysis is blind to the diagnostic group; and that principal component analysis does not cluster participants into separate groups but provides relative weights that indicate the degree to which a participant manifests the relevant clinical features.

**Table 2:** Varimax-rotated component matrix from principal component analysis

	Syndrome dimension 1	Syndrome dimension 2	Syndrome dimension 3	Syndrome dimension 4	Syndrome dimension 5	Syndrome dimension 6
Disinhibition	**0.7399**	0.0774	−0.0790	−0.1423	−0.1576	0.1102
Apathy	**0.5486**	0.0763	**0.4276**	0.1221	−0.1919	0.1450
Loss of sympathy or empathy	**0.7022**	0.1278	0.0044	−0.0981	−0.1278	0.0205
Stereotyped/compulsive behaviour	**0.5789**	0.2890	−0.3103	−0.1798	−0.0536	0.2444
Hyperorality/dietary change	**0.6234**	0.0459	−0.2198	−0.1270	−0.0932	0.0705
Executive dysfunction	**0.5458**	0.1440	0.1176	−0.0176	−0.0476	0.3155
CBIR—Abnormal behaviour	**0.7497**	0.1251	−0.0348	−0.0545	−0.0898	−0.3164
CBIR—Mood	**0.5485**	0.1013	0.0039	0.1709	−0.0077	−**0.5067**
CBIR—Eating habits	**0.7647**	0.0394	−0.0486	−0.0500	−0.0074	−0.2188
CBIR—Sleep	**0.4569**	−0.0259	0.2813	0.1776	−0.0488	−**0.4043**
CBIR—Motor behaviour	**0.7056**	−0.0161	−0.1997	−0.1174	0.0726	−0.2513
CBIR—Motivation	**0.7075**	0.2981	0.1511	0.0500	−0.1157	−0.1488
ACER—Attention/orientation	−0.1510	−**0.9002**	0.0922	0.0463	−0.0248	0.0724
ACER—Memory	−0.1124	−**0.8258**	0.3410	0.1746	−0.0440	0.0770
ACER—Fluency	−0.1784	−**0.7576**	0.1183	0.1772	−0.0744	−0.1227
ACER—Language	−0.0805	−**0.8460**	0.3405	0.1337	0.0314	0.0238
ACER—Visuospatial	−0.0532	−**0.8299**	−0.1642	−0.1818	−0.0460	0.1518
CBIR—Memory	**0.4864**	**0.5544**	−0.2152	−0.0392	0.0199	−0.3158
CBIR—Everyday skills	**0.4086**	**0.5214**	0.3309	0.3198	0.0727	−0.1291
Symmetrical parkinsonism	0.0127	−0.0415	**0.7676**	−0.3673	0.0655	−0.0362
Axial rigidity	−0.0156	−0.1158	**0.8077**	0.0425	−0.0231	0.0669
Poor levodopa responsiveness	−0.1307	−0.1057	**0.6757**	0.0873	−0.0629	−0.0536
Postural instability	−0.0504	−0.1069	**0.7719**	0.1690	−0.0640	−0.0241
Supranuclear gaze palsy	−0.0938	−0.1144	**0.8132**	0.1045	−0.0473	0.0526
CBIR—Self care	0.3459	0.3996	**0.4524**	0.3626	−0.0628	−0.0775
Impaired semantics	0.1510	0.3067	−**0.5187**	−0.2377	0.0353	0.1970
Asymmetrical parkinsonism	−0.0652	−0.0843	0.0700	**0.8343**	−0.0627	0.0202
Asymmetrical dystonia	0.0282	−0.0673	0.0899	**0.8300**	−0.0550	0.1061
Asymmetrical myoclonus	−0.0340	−0.0148	−0.0493	**0.6830**	0.1012	0.0621
Limb apraxia	−0.1292	−0.0590	0.1252	**0.5274**	**0.5056**	−0.0233
Cortical sensory loss	−0.1462	−0.0201	0.0584	**0.5635**	0.2569	−0.2505
Alien limb syndrome	−0.0363	−0.0066	0.0504	**0.5423**	0.0749	−0.1367
Symmetrical myoclonus	−0.0658	−0.0350	0.0044	−0.0093	**0.5132**	−0.3228
Agrammatic, apraxic speech	−0.1224	0.1231	−0.0369	0.1137	**0.7667**	0.2703
Logopenic speech	−0.0659	0.0268	−0.1180	−0.0461	**0.7752**	0.0415
CBIR—Beliefs	0.1830	0.2358	0.0220	−0.0019	0.0093	−**0.5919**
Symmetrical dystonia	0.1134	0.1176	0.3325	−0.1741	0.2010	0.0008
Orobuccal apraxia	−0.1225	−0.0170	−0.0012	0.2822	0.3727	−0.0267
Visuospatial deficits	−0.1863	0.1862	−0.0106	0.2386	0.3650	−0.2559
Motor neuron disease	0.2615	−0.1304	−0.1584	−0.0617	−0.1683	0.0556

Positive loadings indicate worse performance or presence of symptoms, except for ACER where negative loadings indicate worse performance. Factor loadings above 0.4 or below −0.4 shown in bold.

ACER, Addenbrooke’s Cognitive Examination - Revised; CBIR, Cambridge Behavioural Inventory - Revised.

There is a range of scores across FTLD subgroups in each of the symptom domains, for each diagnostic group (see online supplemental figures S1 and S2). Syndrome dimension 1 reflected high clinician and carer rating of behavioural impairment. Syndrome dimension 2 reflected cognitive impairment, with contribution from all ACER subscales and carer ratings of memory and everyday skills. Syndrome dimension 3 mirrored a PSP-RS-like motor phenotype, with positive loadings reflecting symmetrical parkinsonism, falls and supranuclear gaze palsy. Negative loadings on this dimension reflected semantic language impairment. The fourth syndrome dimension represented asymmetrical parkinsonism, myoclonus and dystonia with cortical features of alien limb syndrome, apraxia and cortical sensory loss. Syndrome dimension 5 was driven by language impairments including speech apraxia, loss of syntactic comprehension and impaired repetition. Syndrome dimension 6 reflected carer ratings of low mood and abnormal beliefs.

10.1136/jnnp-2020-324903.supp1 Supplementary data

**Figure 3:**
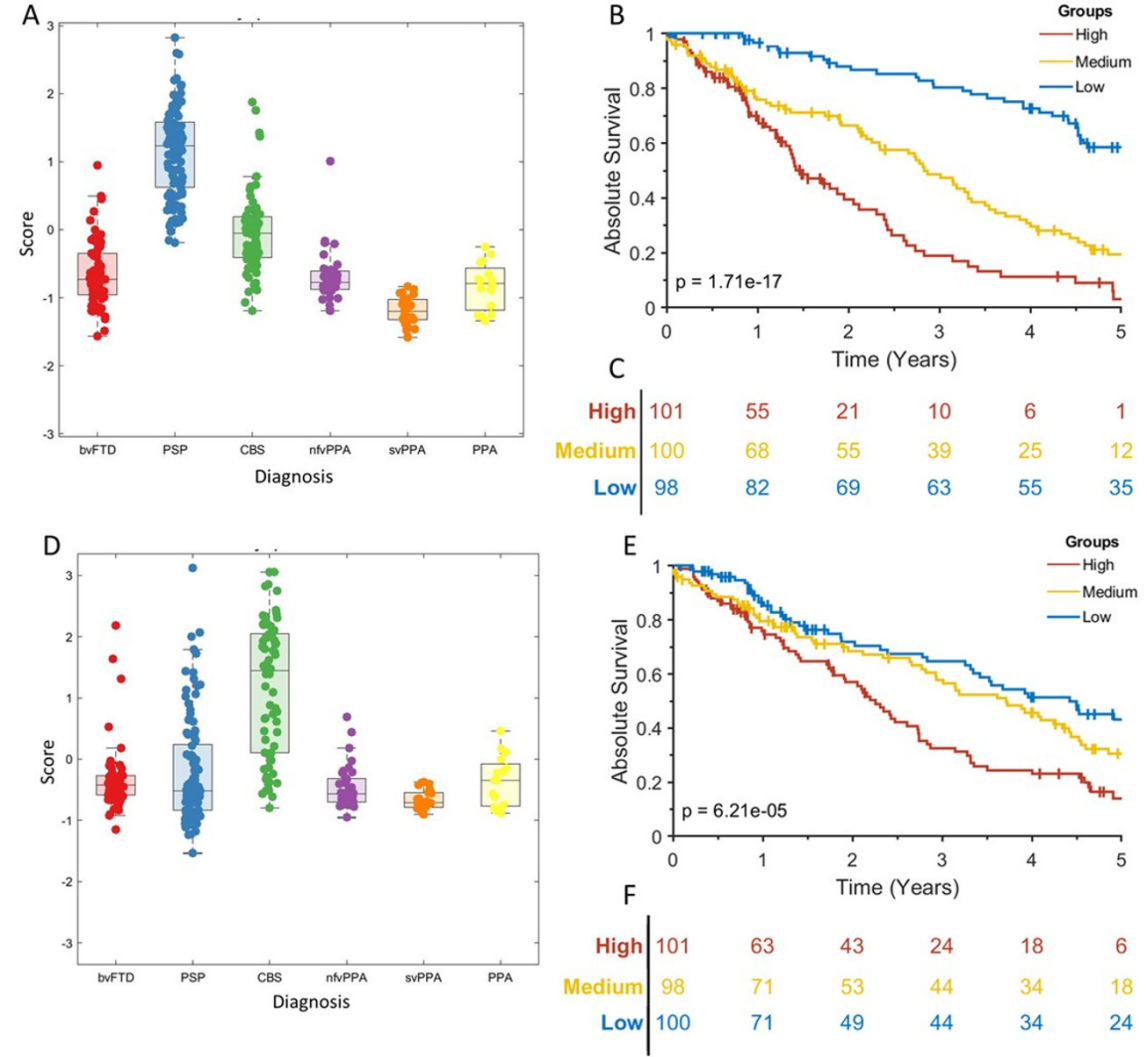
Absolute survival (time to death) in frontotemporal lobar degeneration syndromes. (A) Scatter box plot of individual’s scores on syndrome dimension 3, grouped by frontotemporal lobar degeneration (FTLD) syndrome subtype. The p value is from a log-rank test of the null hypothesis of no difference in survival between all groups. Vertical lines show censored data. (B) KaplanMeier survival curve for high, medium and low scoring tertiles for syndrome dimension 3. (C) At-risk table for the data shown in (B). (D) Scatter box plot of individual’s scores on syndrome dimension 4. (E) Kaplan-Meier survival curve for high, medium and low scoring tertiles for syndrome dimension 4. (F) At-risk table for the data shown in (E). bvFTD, behavioural variant frontotemporal dementia; CBS, corticobasal syndrome; nfvPPA, non-fluent variant primary progressive aphasia; PPA, progressive aphasia; PSP, progressive supranuclear palsy; svPPA, semantic variant primary progressive aphasia.

**Figure 4:**
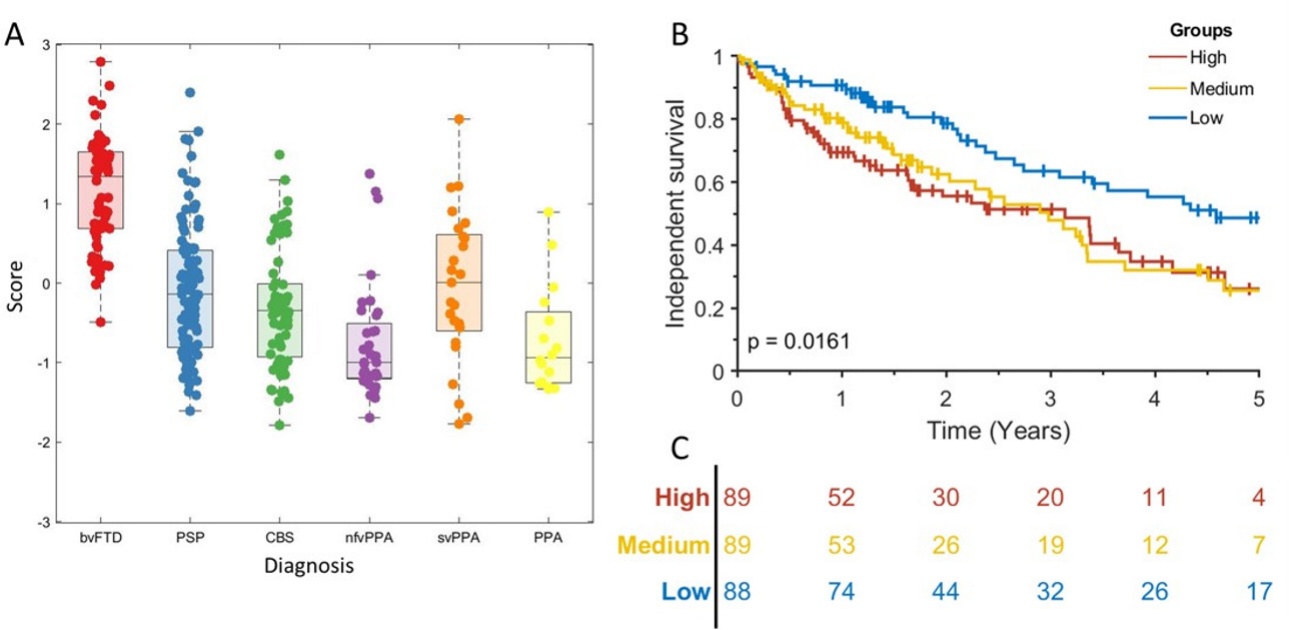
Independent survival (time to care home admission) in frontotemporal lobar degeneration syndromes. (A) Scatter box plot of each participant’s score on syndrome dimension 1. (B) Kaplan-Meier survival curve for high, medium and low scoring tertiles for syndrome dimension 1. The p value is from a log-rank test of the null hypothesis of no difference in survival between all groups. Vertical lines show censored data. (C) At-risk table for the data shown in (B). bvFTD, behavioural variant frontotemporal dementia; CBS, corticobasal syndrome; nfvPPA, non-fluent variant primary progressive aphasia; PPA, progressive aphasia; PSP, progressive supranuclear palsy; svPPA, semantic variant primary progressive aphasia.

Cox proportional hazards regression indicated that syndrome dimensions 3 and 4 and age at clinical assessment were associated with shorter time to death (table 3). Syndrome dimension 3 remained a significant predictor of death after PSP was removed (HR 2.30, CI 1.50 to 3.52, p<0.001). Absolute survival (time from assessment to death) differed between participants in high, medium and low severity tertiles for syndrome dimensions 3 (figure 3A) and 4 (figure 3B) severity score. This effect persisted after removing the highest scoring FTLD subgroups, PSP for syndrome dimension 3 (log rank p<0.001) and CBS for syndrome dimension 4 (log rank p<0.001).

**Table 3:** Cox proportional hazards model of time from study assessment to death.

	Hazard Ratio	CI	Coefficient	SE	P value
**Age**	1.04	1.02 to 1.06	0.04	0.01	**<0.01**
Gender	1.18	0.87 to 1.59	0.16	0.16	0.29
Diagnosis 1	0.64	0.23 to 1.80	−0.45	0.53	0.40
Diagnosis 2	0.87	0.48 to 1.57	−0.14	0.3	0.64
Diagnosis 3	1.16	0.53 to 2.54	0.15	0.4	0.71
Diagnosis 4	0.99	0.5 to 1.97	−0.01	0.35	0.97
Diagnosis 5	0.65	0.25 to 1.64	−0.44	0.48	0.36
Syndrome dimension 1	1.23	0.98 to 1.55	0.21	0.12	0.07
Syndrome dimension 2	1.15	0.99 to 1.35	0.14	0.08	0.07
**Syndrome dimension 3**	1.97	1.41 to 2.75	0.68	0.17	**<0.01**
**Syndrome dimension 4**	1.31	1.07 to 1.61	0.27	0.1	**<0.01**
Syndrome dimension 5	0.87	0.73 to 1.03	−0.14	0.09	0.12
Syndrome dimension 6	0.88	0.75 to 1.03	−0.13	0.08	0.12

P values <0.05 in bold.

CI, Confidence interval of the hazard ratio; SE, Standard error.

Next, we tested which syndrome dimensions predicted care home admission at 2 years with age, gender and disease group as covariates. Eighty-nine patients were excluded from this analysis due to incomplete follow-up. Syndrome dimension 1, reflecting behavioural impairment, was associated with care home admission (OR 2.46, p<0.001) (table 4). This remained a significant predictor of care home admission even after bvFTD, the subgroup with highest scores, was removed (OR 3.20, p=0.03). Independent survival (time from clinical assessment to care home admission or death) differed between participants in high, medium and low severity tertiles for syndrome dimension 1 severity score (log-rank p=0.007) (figure 4). This result persisted after removing the bvFTD group (log-rank p<0.001).

**Table 4:** Logistic regression of predictors of care home admission by 2 years from clinical assessment

	Odds Ratio	Coefficient	t value	P value
Constant	0.10	−2.26	−1.17	0.24
Age	1.02	0.02	0.79	0.43
Gender	0.78	−0.25	−0.60	0.55
Diagnosis 1	0.79	−0.23	−0.20	0.84
Diagnosis 2	2.89	1.06	1.40	0.16
Diagnosis 3	0.50	−0.70	−0.72	0.47
Diagnosis 4	0.13	−2.02	−1.54	0.12
Diagnosis 5	0.28	−1.28	−1.07	0.28
**Syndrome dimension 1**	2.46	0.90	3.11	**<0.01**
Syndrome dimension 2	1.42	0.35	1.60	0.11
Syndrome dimension 3	1.13	0.12	0.28	0.78
Syndrome dimension 4	0.99	−0.01	−0.03	0.98
Syndrome dimension 5	1.08	0.08	0.36	0.72
Syndrome dimension 6	0.77	−0.26	−1.20	0.23

P values <0.05 in bold.

## Discussion

The principal result of this study is that clinician-rated and carer-rated behavioural disturbance are associated with shorter functionally independent survival, while the presence of motor features (including parkinsonism, postural instability, supranuclear gaze palsy, dystonia and apraxia) is associated with reduced absolute survival. These associations are found across the spectrum of common syndromes associated with FTLD, even when groups classically associated with these clinical features are excluded (bvFTD and PSP/CBS, respectively). The participants’ weightings on the syndrome dimensions from our analysis predicted mortality and care home admission better than their diagnostic group, as defined by current consensus diagnostic criteria. We suggest that a transdiagnostic approach that captures the clinical overlap and mixed phenotype adds clinically relevant information for prognostication to that available from the diagnostic group label.

Behavioural impairment, represented here by syndrome dimension 1, was associated with a greater risk of care home admission. This complements previous findings in bvFTD,^4^ Parkinson’s and Alzheimer’s disease.^23 24^ Syndrome dimension 1 reflects behavioural impairments including apathy, impulsivity, socially inappropriate behaviour and hyperorality. More detailed neuropsychological tests and measures of carer burden could fractionate behavioural impairment to more closely determine which behavioural impairments have the greater effect on prognosis.^8 25^ These results show correlation and not causation, and we lack data on the reasons given for care home admission. Behavioural impairments in frontotemporal dementia and PSP increase carer burden^26^ and there are no proven effective pharmacological treatments. Patients with more severe behavioural impairments may require continuous supervision, which becomes difficult for spouses or families to sustain at home. We suggest that treating behavioural disturbance may delay the need for care home admission, with benefits to individual health and health economics. Potential strategies include restoration of neurotransmitter deficits associated with behavioural change^27–30^ or motor impairment.^31^

The relationship between cognitive impairment and prognosis is complex. Some studies show a clear association,^9^ but others do not.^8 32^ This discrepancy may be due to the indirect contribution of behavioural and motor impairments to performance on ‘cognitive’ tests. For example, speech or constructional deficits in nfvPPA or CBS, respectively, may impair performance on tasks that require speaking, writing or drawing. However, the separation of cognitive and motor deficits across the six dimensions argues against such a simple interference effect.

The clinical phenotypes reflected by syndrome dimensions 3 and 4 are classically associated with PSP Richardson’s syndrome and CBS, respectively; in our cohort, however, these dimensions were also expressed to a subtler degree by many other patients except for those with svPPA (figure 2C, D). PSP-RS typically has a worse prognosis than bvFTD (unless there is coexistent MND) and PPA^3 8 33^, while FTLD-tau has a worse prognosis than FTLD-TDP43 if clinical MND cases are excluded.^34^ With disease progression, many patients with nfvPPA develop the phenotype of PSP or CBS, an adverse prognostic sign.^35^ In keeping with these observations, previous survival analyses of frontotemporal dementia (bvFTD and PPA) have shown that reduced letter fluency, motor cortex atrophy and brainstem hypoperfusion were associated with reduced survival.^4 36^ Our results go beyond these findings, suggesting that development of motor impairments, irrespective of diagnostic group, is an adverse prognostic sign. However, the correlation between syndrome dimensions 3 and 4 and mortality does not prove causation. It is unclear whether that these syndrome dimensions are indicative of a more aggressive disease or increased risk of complications, such as aspiration pneumonia due to dysphagia, sarcopenia and other aspects of frailty.^37^ These complications could, in turn, increase mortality.

Our study has limitations. We only included basic covariates in our survival analysis (age, gender and main diagnostic group). Medical and psychiatric comorbidities, marital status, social class, ethnicity and financial status are also known to influence rates of care home admission and death^24^ and may explain some of the variance in prognosis. The use of artificial ventilation and gastrostomy were not prospectively recorded in our cross-sectional study. However, while dysphagia and respiratory failure are likely to confer a high risk of mortality, these treatments might mitigate the risk for those accepting intervention relative to those who refuse. Our study cannot resolve this ambiguity. Motor neuron disease is known to strongly influence survival^10^ but here the presence of MND did not load strongly onto any syndrome dimension. This may be due to low numbers and does not argue against the relevance of MND for prognosis. We attempted to recruit all patients with a designated syndrome associated with FTLD in our catchment area. Most referrals came from secondary care, so survival rates could be overestimated if patients with rapidly progressively disease died before they came to review. However, average survival in our FTLD cohort was similar to those published previously.^3^ We did not distinguish between residential or nursing care from basic demographic information. This was not differentiated in our demographic data because many institutions provide both levels of care at the same site. We also highlight that admission to a residential or nursing home is not a sign of inadequate home care and not inevitably associated with reduced quality of life. Patients can benefit from skilled holistic care provided in these institutions. However, at a group level care home admission is a measure of reduced independence, and a potential study endpoint in trials. We did not include functional rating scales in our analysis. Such scales provide relevant patient-centred endpoints, but they may be inconsistent when applied across the full spectrum of FTLD disorders, or reflect carer health and support, and be weighted towards subsets of features. We acknowledge that although care home admission is a definite endpoint, it is not a direct measure of functional dependence. Further research on individual risk factors of survival in FTLD is required to identify which specific features within the syndrome dimensions most strongly predict death and care home admission. Finally, our UK-based data may have limited applicability to countries with differences in ethnicity, medical-care and social-care practices.

Our results have implications for the clinical treatment of patients with FTLD syndromes. They suggest that the association between diagnosis or ‘proteinopathy’ and survival is weak, with the caveat that only a subset of our cohort has a neuropathological diagnosis at the time of publication. Instead, survival was more closely associated with phenotypic features across the spectrum of FTLD syndromes. To halt or reverse the neurodegeneration caused by FTLD is a long-term goal. However, treating symptoms irrespective of diagnosis and aetiology remains important, for example, by targeting common neurotransmitters deficits.^27^ Such treatments could improve patients’ quality of life and may also improve survival, analogous to the effect of levodopa in Parkinson’s disease.

In summary, functionally independent and absolute survival in syndromes associated with frontotemporal lobar degeneration are predicted by a subset of clinical features, over and above the diagnostic label. Given these findings, and the overlapping clinical,^5 7^ structural,^38^ functional,^39^ neuropathological^40^ and neurochemical^27^ features in these syndromes, we recommend a transdiagnostic approach to develop better treatment strategies. Effective treatments for behavioural and motor features could improve functionally independent survival and might reduce absolute mortality.

## Data Availability

Data are available upon reasonable request. Anonymised derived data are available on reasonable request for academic purposes, subject to the protection of personally identifiable data.
