# Supplementary material for: Predicting loss of independence and mortality in frontotemporal lobar degeneration syndromes
Source: J Neurol Neurosurg Psychiatry. 2021 Feb 9;92(7):737–44. doi: 10.1136/jnnp-2020-324903 (PMC8223632; doi:10.1136/jnnp-2020-324903)
Supplement: Supplementary data [file jnnp-2020-324903supp001.pdf]

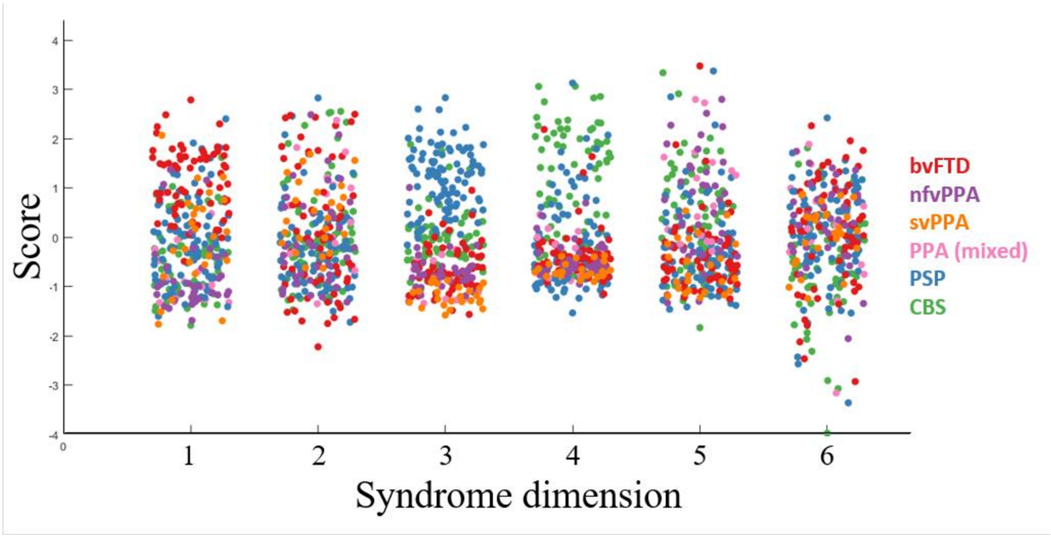

Figure S1: Scatterplot of participant scores on each of the six syndrome dimensions (principal components), colour coded by clinical diagnostic group. Note that every dimension contains every participant, but that participants' positions change. For example in syndrome dimension 3, the svPPA (orange) are and the lower end of the core range in contrast to PSP (blue) at the upper end. In contrast, in syndrome dimension 1, the same svPPA cases are scattered in the mid-range, while bvFTD are mainly towards the upper end and nfvPPA toward the lower end of the score range.

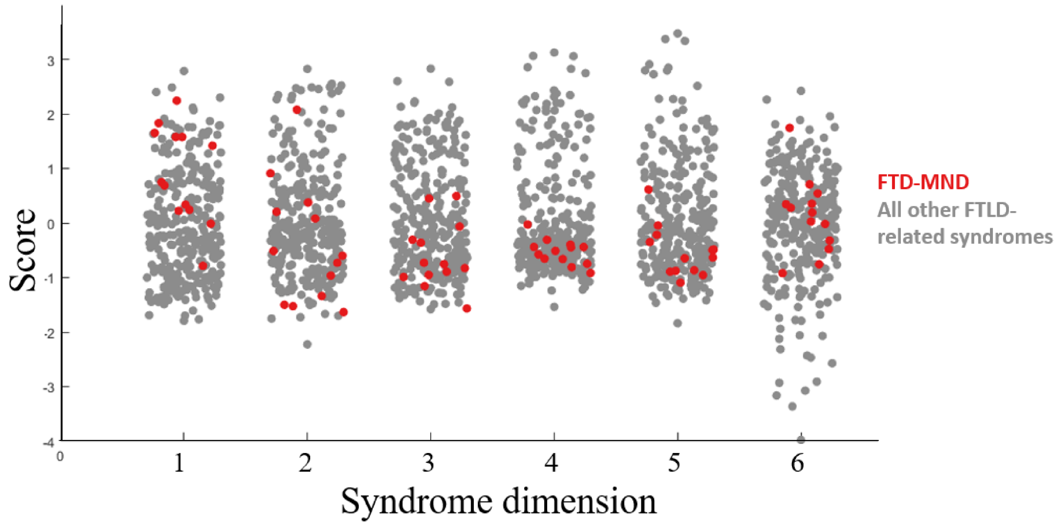

Figure S2: Scatterplot of participants scores on each syndrome dimension. This is the same plot as figure S1 but with the FTD-MND patients highlighted. MND patients without FTD-features were not included in this study.
